# GC/MS Profiling, Anti-Collagenase, Anti-Elastase, Anti-Tyrosinase and Anti-Hyaluronidase Activities of a *Stenocarpus sinuatus* Leaves Extract

**DOI:** 10.3390/plants11070918

**Published:** 2022-03-29

**Authors:** Mai M. Younis, Iriny M. Ayoub, Nada M. Mostafa, Mahmoud A. El Hassab, Wagdy M. Eldehna, Sara T. Al-Rashood, Omayma A. Eldahshan

**Affiliations:** 1 Department of Pharmacognosy, Faculty of Pharmacy, Ain Shams University, Abbassia, Cairo 11566, Egypt; mai.m.younis@pharma.asu.edu.eg (M.M.Y.); irinyayoub@pharma.asu.edu.eg (I.M.A.); nadamostafa@pharma.asu.edu.eg (N.M.M.); 2 Department of Medicinal Chemistry, Faculty of Pharmacy, King Salman International University (KSIU), South Sinai 46612, Egypt; mahmoud65582@pharm.tanta.edu.eg; 3 School of Biotechnology, Badr University in Cairo, Badr City, Cairo 11829, Egypt; wagdy.mohamad@pharm.kfs.edu.eg; 4 Department of Pharmaceutical Chemistry, Faculty of Pharmacy, Kafrelsheikh University, Kafrelsheikh 33516, Egypt; 5 Department of Pharmaceutical Chemistry, College of Pharmacy, King Saud University, P.O. Box 2457, Riyadh 11451, Saudi Arabia; salrashood@ksu.edu.sa

**Keywords:** antiaging, *Stenocarpus sinuatus*, anti-collagenase, anti-elastase, anti-tyrosinase, anti-hyaluronidase, GC/MS

## Abstract

Today, skin care products and cosmetic preparations containing natural ingredients are widely preferred by consumers. Therefore, many cosmetic brands are encouraged to offer more natural products to the market, such as plant extracts that can be used for their antiaging, antiwrinkle, and depigmentation properties and other cosmetic purposes. In the current study, the volatile constituents of the hexane-soluble fraction of a *Stenocarpus sinuatus* (family Proteaceae) leaf methanol extract (SSHF) were analyzed using GC/MS analysis. Moreover, the antiaging activity of SSHF was evaluated through in vitro studies of anti-collagenase, anti-elastase, anti-tyrosinase, and anti-hyaluronidase activities. In addition, an in silico docking study was carried out to identify the interaction mechanisms of the major compounds in SSHF with the active sites of the target enzymes. Furthermore, an in silico toxicity study of the identified compounds in SSHF was performed. It was revealed that vitamin E (*α*-tocopherol) was the major constituent of SSHF, representing 52.59% of the extract, followed by *γ*-sitosterol (8.65%), neophytadiene (8.19%), *β*-tocopherol (6.07%), and others. The in vitro studies showed a significant inhibition by SSHF of collagenase, elastase, tyrosinase, and hyaluronidase, with IC_50_ values of 60.03, 177.5, 67.5, and 38.8 µg/mL, respectively, comparable to those of the positive controls epigallocatechin gallate (ECGC, for collagenase, elastase, hyaluronidase) and kojic acid (for tyrosinase). Additionally, the molecular docking study revealed good acceptable binding scores of the four major compounds, comparable to those of ECGC and kojic acid. Besides, the SSHF identified phytoconstituents showed no predicted potential toxicity nor skin toxicity, as determined in silico. In conclusion, the antiaging potential of SSHF may be attributed to its high content of vitamin E in addition to the synergetic effect of other volatile constituents. Thus, SSHF could be incorporated in pharmaceutical skin care products and cosmetics after further studies.

## 1. Introduction

Skin aging is a complex biological process. It causes various undesirable visible signs such as skin dryness, wrinkles, fine lines, reduction in skin elasticity, and loss of skin firmness and soft texture [1]. It develops as a result of both intrinsic and extrinsic factors. The intrinsic factors include genetic, hormonal, and cellular metabolic factors. The extrinsic factors are the result of chronic exposure to agents with damaging effects to the skin such as toxins, chemicals, nicotine, pollutants, and, particularly, exposure to sunlight radiation, especially UV-B, which causes photoaging and skin cancer [2]. These factors can lead to an increase in matrix metalloproteinases (MMPs) expression in human skin. MMPs are responsible for senescent cells accumulation, connective tissue degradation, and elastic fibers degradation [3].

Collagenase and elastase are members of the MMPs family that degrade collagen network and breakdown elastin fibers, respectively. As a result, wrinkles appearance, loss of skin elasticity, and consequently skin aging occur [4]. Tyrosinase is the rate-limiting enzyme in melanin synthesis, and its overproduction results in melanin accumulation and various skin disorders, including hyperpigmentation, sagging, wrinkles, freckles, and age spots. Hyaluronidase degrades hyaluronic acid, which is necessary to retain water and keep the skin tissues moist, smooth, well hydrated, and lubricated [5]. Thus, the breakdown of hyaluronic acid by hyaluronidase results in skin drying and sagging and loss of skin smoothness and plasticity [6]. Therefore, medical and cosmetic preparations used to protect skin against aging and hyperpigmentation are mainly composed of collagenase, tyrosinase, elastase, and hyaluronidase inhibitors [4].

However, synthetic ingredients used in cosmetics and medical preparations are not widely accepted by consumers, due to their undesirable side effects and allergic reactions. As a consequence, cosmetic research has extended to identify natural resources for cosmetics [6]. Many natural products have shown potential antioxidant, anti-inflammatory [7,8,9], antiaging, and anti-hyperpigmentation effects. Green tea (*Camellia* *sinensis*) extracts, grape (*Vitis vinifera*) seed oil, soya bean (*Glycine max*) extracts, and coconut (*Cocos nucifera*) oil are good examples of natural antiaging products [6].

Natural products are considered as a reservoir for treating and combating many diseases [10,11,12,13,14,15,16,17,18,19,20]. The family Proteaceae is considered one of the important medicinal plant families owing to the different significant biological activities exerted by its family members. For example, the stem and bark extracts of *Faurea saligna* have been reported to exert in vitro antibacterial and antifungal activities. In addition, an in vitro anti-leishmanial effect has been reported for a *Stenocarpus sinuatus* bark extract against *Leishmania amazonensis promastigotes.* These significant activities have been correlated to the diversity of the classes of compounds isolated from Proteaceous plants. These include flavonoids, alkaloids, terpenoids, sterols, and phenolic compounds. Furthermore, significant activities of the compounds isolated from Proteaceous plants have been described. For example, bisresorcinol isolated from a *Heliciopsis terminalis* trunk extract was reported to exert in-vitro antioxidant, anti-inflammatory, and hepatoprotective effects. In addition, lomatiol isolated from different species of the genus *Lomatia* exhibited cytotoxic activity, and helicid isolated from *Helicia nilagirica* showed potent antidepressant activity in vivo [21,22,23,24,25,26,27,28,29].

Moreover, many plant extracts and oils belonging to the family Proteaceae have been reported to exert significant antiaging, moisturizing, and skin whitening effects. For example, the oil of *Macadamia integrifolia* (Proteaceae) has been incorporated as an antiaging ingredient in cosmetic preparations. In addition, its leaf extract was reported to exhibit potent anti-tyrosinase activity, with IC_50_ of 85 µg/mL, whereas the ethyl acetate and *n*-butanol fractions showed IC_50_ values of 60 and 75 µg/mL, respectively. Besides, gallic acid isolated from this plant exhibited an IC_50_ value 56 µg/mL [30]. These remarkable cosmetic effects of *Macadamia integrifolia* oil and leaf extract have been attributed to their content of vitamin E and phytosterols [31,32]. *Protea madiensis* root and bark extracts have been traditionally used to treat hyperpigmentation and other skin disorders. They were found to inhibit mushroom tyrosinase and tyrosine hydroxylase activity (THA). The *n*-hexane and methanol extracts inhibited the enzyme tyrosinase, with IC_50_ of 40 and 31 μg/mL, respectively. Meanwhile, the *n*-hexane, chloroform, methanol, and aqueous extracts inhibited THA with IC_50_ > 16.7, =74 ± 17, <16, and <52 μg/mL, respectively. Moreover, they strongly inhibited melanogenesis [33]. Additionally, methyl 2,5-dihydroxycinnamate and bis-norstriatol isolated from a methanolic extract of *Grevillea robusta* leaves was reported to inhibit L-DOPA oxidation by mushroom tyrosinase, with IC_50_ values of 69.22 and 65.54 μM, compared to a value of 114.54 μM of a reference standard (kojic acid) [34]. Furthermore, an *Oreocallis grandiflora* hydroalcoholic extract exhibited photoprotective activity against UV B cell death. It showed a sun protection factor (SPF) of 13.56 at 10 µg/mL, compared to 11.82 and 6.21 of 2-ethylhexyl 4-methoxycinnamate and 2-ethylhexyl 4-(dimethyl amino) benzoate positive controls, respectively, at the same concentration [35].

Despite the great diversity of the reported phytoconstituents and biological activities of many Proteaceous plants, several family members have not been explored yet. These members are expected to be beneficial in the field of medicine and are worth to be investigated. The anti-leishmanial activity of *Stenocarpus sinuatus* (A. Cunn.) Endl. (Proteaceae) bark extract was previously described [28]. However, the phytoconstituents and biological activities of its leaf extracts have not been investigated yet. *S. sinuatus* is an Australian tree of medium size and approximately 30 m in height. It grows in tropical and subtropical rainforests. It is sometimes named fire wheel tree or white silky oak due to its ornamental flowers of a bright orange-red color. These flowers produce olfactory cues and ultraviolet markers that attract bees. For example, *Pharohylaeus lactiferus,* a rare endemic bee, mainly prefers *S. sinuatus* [36,37]. The interesting previously mentioned antiaging, moisturizing, and skin whitening effects of some Proteaceous plant extracts and oils have encouraged us to investigate S.S. leaf extracts.

The aim of the current study was to investigate the volatile constituents and the biological activities of a *Stenocarpus sinuatus* extract for the first time. The volatile metabolomic profile of SSHF was characterized by GC/MS. Moreover, the ability of the SSHF extract to inhibit skin aging enzymes (collagenase, tyrosinase, hyaluronidase, and elastase) was evaluated. In addition, in silico docking studies were conducted to demonstrate the mechanism and binding pattern of the four major compounds in SSHF to their targets. Moreover, potential skin toxicity was determined in silico for compounds identified in SSHF.

## 2. Results and Discussion

### 2.1. GC/MS Analysis of the Hexane-Soluble Fraction of a Stenocarpus sinuatus Extract

**Figure 1:**
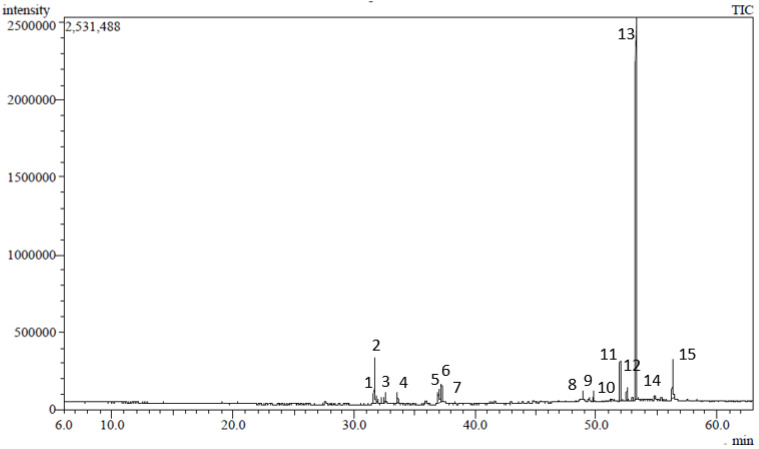
GC/MS chromatogram of *Stenocarpus sinuatus* hexane-soluble fraction.

**Figure 2:**
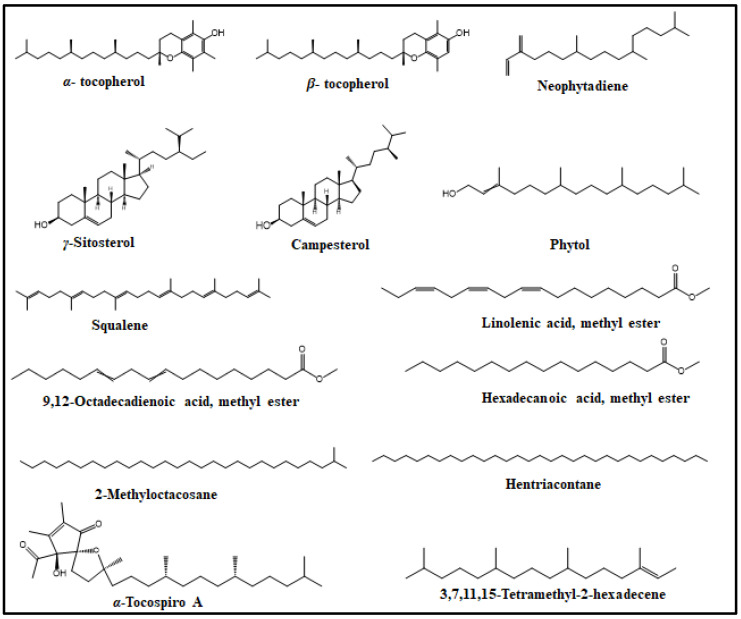
Major constituents of *Stenocarpus sinuatus* hexane-soluble fraction.

The phytochemical profile of the *n*-hexane soluble fraction of a *S. sinuatus* methanol leaf extract was characterized using GC/MS. Fifteen compounds were identified, accounting for 96.77% of SSHF composition (Table 1, Figure 1). Vitamin E (*α*-tocopherol) was the major constituent identified in SSHF, representing 52.59% of the total fraction. Other identified compounds included *γ*-sitosterol (8.65%), neophytadiene (8.19%), *β*-tocopherol (6.07%), linolenic acid, methyl ester (4.84%), and phytol (4.04%). The other identified compounds (Figure 2) belong to different classes including diterpenes, triterpenes, fatty acid methyl esters, aliphatic hydrocarbons, and others.

*α*-Tocopherol (Figure 2), the major constituent identified herein, is the most biologically active isomer of vitamin E [38]. Vitamin E is composed of naturally occurring lipophilic compounds including *α*-, *β*-, *γ*-, and *δ*- tocopherols. They differ in the saturation of their side chains and in the methylation degree of their chromanol heads [39]. It was reported in various plants including leaves of *Sauropus androgynus* (426.8 mg/kg edible part), *Capsicum annum* red pepper (155.4 mg/kg), *Camelia chinensis* black tea (183.3 mg/kg), and many others [40].

Many Proteaceous plants were reported to be rich in tocopherol, phytosterols, squalene, and other constituents. For example, *Macadamia integrifolia* was shown to contain 133.18 mg of vitamin E in 100 g leaves, 2.6% in its kernel, and 5.52% in the pericarp [31]. Macadamia nuts and kernel were reported as rich sources of phytosterols, squalene, and tocopherols [32]. In addition, *Gevuina avellana (Chilean hazelnut*) displayed a high content of vitamin E [41]. Furthermore, the average total tocopherol content of the nut oil of *Gevuina avellana* was 1.4 µg/g [42].

### 2.2. In Vitro Assays to Evaluate the Antiaging Potential of Stenocarpus sinuatus Leaf Hexane-Soluble Fraction

**Figure 3:**
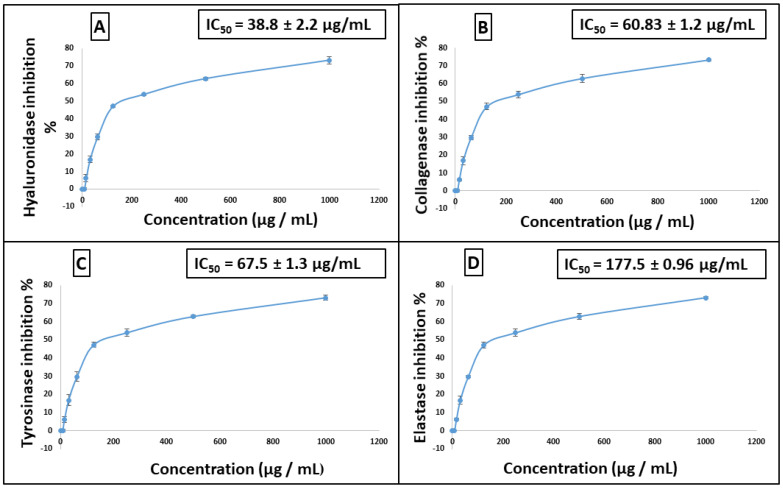
(**A**) Dose–response curve of SSHF inhibition of hyaluronidase, (**B**) collagenase, (**C**) tyrosinase, (**D**) elastase activity. All determinations were carried out in triplicate, and the values are expressed as mean ± SD.

The antiaging potential of *Stenocarpus sinuatus* leaf hexane-soluble fraction was evaluated in vitro. The results revealed a promising activity of SSHF against several enzymes. Different concentrations of the sample were assessed and showed a dose-dependent inhibitory activity against four enzymes (Figure 3). SSHF displayed considerable anti-hyaluronidase and anti-collagenase activities, exhibiting IC_50_ of 38.8 and 60.03 µg/mL, respectively, approaching that of the standard anti-aging drug epigallocatechin gallate (EGCG) with IC_50_ values of 15.5 and 24.7 µg/mL, respectively. In addition, SSHF inhibited tyrosinase, with IC_50_ value of 67.5 µg/mL, whereas the kojic acid standard had an IC_50_ value of 13.8 µg/mL. Meanwhile, it displayed a lower anti-elastase activity, with IC_50_ of 177.5 µg/mL, whereas the IC_50_ of the EGCG standard was 18.2 µg/mL.

The identified constituents of SSHF were reported to exert significant biological activities useful in the cosmetic field. Their antiaging, skin whitening, skin moisturizing, and antioxidant effectiveness was observed. These reported beneficial activities encouraged us to evaluate the antiaging potential of the isolated fraction through different in vitro and molecular docking assays.

For example, vitamin E (52.59%) is well known for its antioxidant, free radicle scavenging, and antiaging activities [38]. Moreover, it reduces ultraviolet-induced skin hyperpigmentation. Therefore, its cosmetic application protects the skin from UV damage that aggravates wrinkles, skin dehydration, and loss of elasticity [43]. The depigmentation effect of vitamin E was attributed to its reported anti-tyrosinase and antioxidant activities [44].

Several studies were previously conducted to illustrate the role of vitamin E in the inhibition of the pro-aging enzymes assessed herein. An in vitro study with skin fibroblasts revealed that *α*-tocopherol reduced the level of expression of collagenase via protein kinase C inhibition. It diminished the transcription of MMP-1 mRNA at 50 µM. This inhibition explained the effect of *α*-tocopherol against skin age-dependent damage and inflammation [45]. Vitamin E was also used as a reference standard in antioxidant and anti-tyrosinase assays, showing a DPPH scavenging effect with an IC50 of 25.55 µg/mL and inhibition of tyrosinase with a percentage of inhibition of 36.27 ± 3.73% [46].

An in vivo study on 10 female volunteers examined the effectiveness of a vitamin E microsphere formulation. Enhancement of skin elasticity and skin moisture and decrease of wrinkles volume were reported [47]. Consequently, vitamin E can be used either alone or in combination with other ingredients in cosmetic preparations to improve skin integrity and pigment appearance besides reducing skin aging [43]. Thus, the high content of vitamin E in SSHF suggests a significant antiaging activity of this extract.

Phytosterol constituents identified in SSHF such as *γ*-sitosterol (8.65%) and Campesterol (0.66%) were reported to be biologically active, exhibiting emollient and antiaging effects and protecting the skin against UV damage. Moreover, the ability of phytosterols to inhibit MMP-1, constrain collagen degradation, and enhance collagen synthesis was experimentally demonstrated in human keratinocytes [48]. Besides, they were reported to stimulate the synthesis of hyaluronic acid, increase the thickness of the epidermis, enhance skin elasticity, and reduce skin roughness. Therefore, they can be incorporated in antiaging creams as well as sun-care products [49].

In addition, many diterpenes isolated from plants have been reported to show potential antioxidant and anti-inflammatory activities [50,51]. Phytol diterpene identified in SSHF (4.04%) was used in cosmetics to inhibit cellular senescence, in particular of keratinocytes, induced by oxidative stress [52]. In It also exhibited antioxidant, anti-inflammatory, antimicrobial, cytotoxic, and immune-modulating activities [53]. Previous molecular docking studies revealed that phytol can bind to amino acid residues in the catalytic domain of MMP-1 (collagenase enzyme), with binding energy (−7.06 kcal/mol) comparable to that (−8.05 kcal/mol) of the reference compound doxycycline [54].

Neophytadiene diterpene (8.19%) exhibited in vitro antioxidant, anti-inflammatory, antipyretic, antimicrobial, and analgesic activities [55]. It showed potent binding affinity to tyrosinase in molecular docking studies, with a docking score of 56.99 [56], while squalene triterpene (1.03%) exhibited in vitro antiaging, sunscreen, antioxidant, anti-inflammatory, and antidermatitic effects [55,57].

Therefore, in the current study, the observed significant activities of SSHF can be explained by the antiaging, skin whitening, and antioxidant effects previously reported for its identified components. Thus, it can be concluded that the significant inhibitory activity of SSHF to hyaluronidase collagenase, tyrosinase, and elastase may be attributed to its high content of vitamin E and the synergetic effects of all its constituents.

### 2.3. In Silico Studies

#### 2.3.1. In Silico Docking Studies on the Target Enzymes

**Figure 4:**
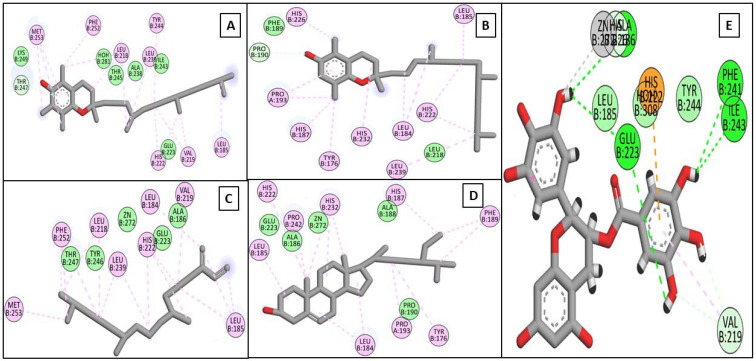
2D binding modes of *α*-tocopherol (**A**), *β*-tocopherol (**B**), neophytadiene (**C**), *γ*-sitosterol (**D**), and EGCG (**E**) to the active binding sites of collagenase.

**Figure 5:**
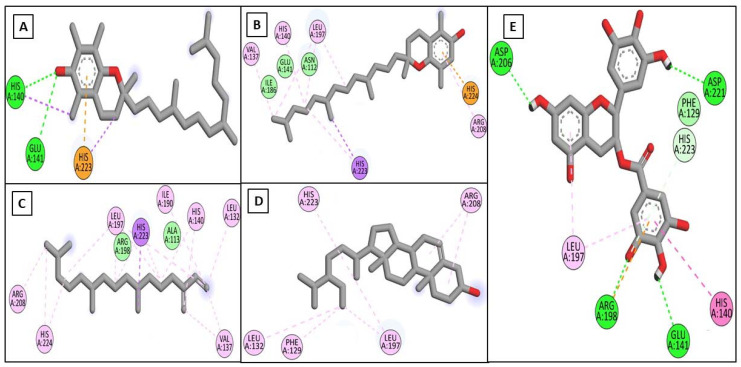
2D binding modes of *α*-tocopherol (**A**), *β*-tocopherol (**B**), neophytadiene (**C**), *γ*-sitosterol (**D**), and EGCG (**E**) to the active binding sites of elastase.

**Figure 6:**
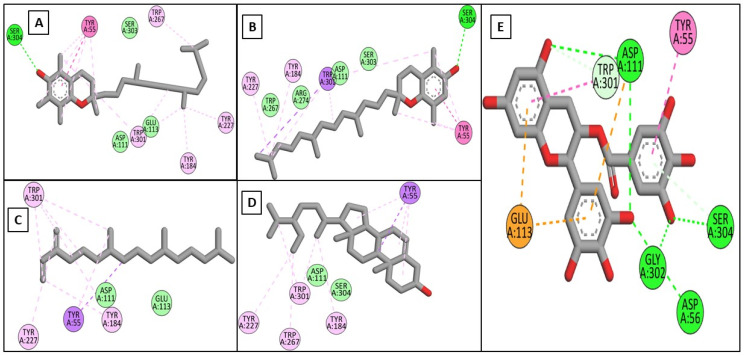
2D binding modes of *α*-tocopherol (**A**), *β*-tocopherol (**B**), neophytadiene (**C**), *γ*-sitosterol (**D**), and EGCG (**E**) to the active binding sites of hyaluronidase.

**Figure 7:**
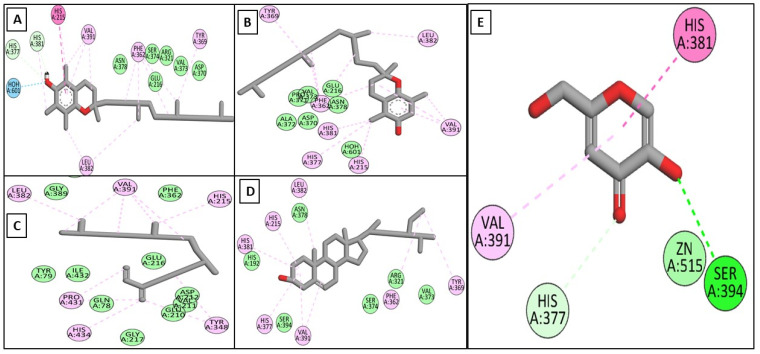
2D binding modes of *α*-tocopherol (**A**), *β*-tocopherol (**B**), neophytadiene (**C**), *γ*-sitosterol (**D**), and kojic acid (**E**) to the active binding sites of tyrosinase.

Driven by the promising inhibitory activity of the extract against collagenase, elastase, hyaluronidase, and tyrosinase, an in silico docking study was conducted. Docking studies are performed to identify the possible binding mechanism and pattern of compounds to potential targets [58,59]. The main compounds that we identified were previously reported to exert antiaging activity in several studies. For example, vitamin E was found to inhibit tyrosinase and collagenase in vitro [45,46]. In addition, neophytadiene showed potent binding to tyrosinase in a previous in silico study [56]. Furthermore, phytosterols were reported to enhance the synthesis of collagen and hyaluronic acid [48,49]. Therefore, we assumed that the major identified compounds might be responsible for the observed activities of SSHF. In this respect, the X-ray structures of the four enzymes were downloaded from the PDB to conduct the docking assays. First, the applied docking parameters were validated by re-docking each o-crystalized ligand into its corresponding active site. The calculated RMSD values between the docked pose and the co-crystalized pose were 0.77, 1.2, 0.61, and 0.81 Å for collagenase, elastase, hyaluronidase, and tyrosinase, respectively, indicating that the docking protocol was valid. The docking of EGCG to hyaluronidase, collagenase, and elastase resulted in docking scores of −8.19, −8.8, and−8.9 Kcal/mole, while the docking of kojic acid to its target enzyme resulted in a docking score of −4.62 Kcal/mole. The docking of the four major compounds to the four enzymes resulted in good acceptable scores, comparable to those of the reference compounds EGCG and kojic acid. Table 2 summarizes the docking interactions of the four compounds with the four potential target enzymes and the corresponding docking scores. Taking into account the hydrophobic nature of the isolated compounds composed mainly of a hydro-carbonic skeleton, most of the observed interactions with the four enzymes were found to be hydrophobic in nature (see Figure 4, Figure 5, Figure 6 and Figure 7). In conclusion, from the major extract, the four identified compounds had the ability to strongly interact with the four enzymes collagenase, elastase, hyaluronidase, and tyrosinase, achieving acceptable docking scores that sometimes exceeded those of the reference compounds. These acceptable scores were achieved through the establishment of many hydrophobic interactions. Thus, the observed strong binding interactions validated the activities of SSHF and suggest possible mechanisms of action.

#### 2.3.2. In Silico Toxicity Study

The SSHF constituents showed no toxicity in silico using Pro-toxll for the prediction of any potential toxicity. In addition, no potential skin toxicity could be predicted when using Pred-Skin 3 and a Bayesian model. The results are shown in Table 3. Furthermore, no reported harmful or toxic effects could be established for any of the compounds identified in SSHF.

## 3. Materials and Methods

### 3.1. Plant Material

The leaves of *Stenocarpus sinuatus* were collected in Spring 2020 from El-Abd farm, Cairo Alexandria desert road, Egypt. Plant material was kindly identified and authenticated by Mrs. Treiz Labib, Plant Taxonomy Consultant at the Ministry of Agriculture, Egypt. A voucher specimen (PHG-P-SS-326) was deposited in the Herbarium of the Pharmacognosy Department, Faculty of Pharmacy, Ain Shams University.

### 3.2. Chemicals and Reagents

Tricine buffer, collagenase (ChC—EC.3.4.23.3), N-[3-(2-furyl) acryloyl]-Leu–Gly–Pro–Ala (FALGPA), EGCG, N-Succinyl-Ala–Ala–Ala-p-nitroanilide (AAAPVN), Tris-HCL buffer, pancreatic elastase (PE), L-DOPA, mushroom tyrosinase, phosphate buffer, Kojic acid, calcium chloride, hyaluronic acid, potassium metaborate (KBO_2),_ hyaluronidase, acetate buffer, 10 N HCl, acetic acid, and p-dimethylaminobenzaldehyde (DMAB) were bought from Sigma-Aldrich (Heliopolis, Cairo, Egypt). Methanol and hexane were bought from Al-brouj (Giza, Egypt). All solvents used were of analytical grade.

### 3.3. Preparation of the Extracts

The air-dried leaves were ground and extracted with methanol (12 L × 3). The extract was filtered, and the solvent was evaporated using a rotary evaporator under reduced pressure and lyophilized to yield 350 g of methanol extract. The extract was then defatted with hexane (3 L × 3). The hexane soluble fraction was evaporated under reduced pressure to yield 45 g of residue. The hexane soluble fraction was kept in a tightly closed container for further analysis.

### 3.4. GC/MS Analysis of the Hexane-Soluble Fraction

GC/MS analysis of the hexane-soluble fraction was carried out using a Shimadzu QP2010 gas chromatograph coupled to a quadrupole mass spectrometer (Shimadzu Corporation, Kyoto, Japan) [60,61,62] and equipped with an Rtx-5MS fused bonded capillary column (Restek, PA, USA, dimensions, 30 m × 0.25 mm i.d. × 0.25 μm film thickness) and a split–splitless injector. Helium was used as a carrier gas at a flow rate of 1.37 mL/min, and an injection volume 1 μL of the diluted sample (1% *v*/*v*) was employed in split mode at a split ratio of 1:15. The injector temperature was adjusted at 280 °C, the oven temperature was kept at 50 °C for 3 min, then programmed to reach 300 °C at a rate of 5 °C/min and kept constant at 300 °C for 5 min. As the MS operating parameters, the ion source temperature was maintained at 220 °C, and the separation was carried out at 70 eV in electron ionization (EI) mode, filament emission current 60 mA, and scanning from 35 to 500 amu. The compounds were identified by the comparison of their retention indices (RI) and mass spectra (MS) to values reported in the NIST mass spectral library database (similarity index > 90%) and in the literature [63,64,65,66,67,68,69,70,71,72]. The retention indices were calculated relative to those of a homologous series of standard n-alkanes (C8–C28) injected under the same conditions. The results was processed by GCMSsolution Workstation Software for Gas Chromatography–Mass Spectrometry.

### 3.5. In Vitro Antiaging Assays

#### 3.5.1. Anti-Collagenase Assay

The anti-collagenase assay was performed spectrophotometrically according to the method reported by Thring et al. [73] with minor modifications. The assay was carried out in 50 mM Tricine buffer (pH 7.5 with 10 mM CaCl_2_ and 400 mM NaCl). Collagenase produced by the bacterium *Clostridium histolyticum* (ChC—EC.3.4.23.3) was first dissolved in the buffer to an initial concentration of 0.8 unit/mL based on the supplier’s activity data. N-[3-(2-furyl) acryloyl]-Leu–Gly–Pro–Ala (FALGPA) was used as a synthetic substrate, dissolved in Tricine buffer to a concentration of 2 mM. The samples in a concentration range of 1000–7.81 µg/mL were incubated at room temperature with the prepared collagenase in Tricine buffer for 15 min; then, the synthetic substrate was added to the samples to start the reaction. The absorbance values were measured at 490 nm using a microplate reader (TECAN, Inc., Durham, NC, USA). A positive control (EGCG) was used, while the negative control consisted of water. The percentage of collagenase inhibition was calculated according to (%) = [1 − (S/C) × 100], where “S” is the corrected absorbance of the controls (in the absence of a sample). The inhibitory concentration 50, inhibiting 50% of the enzyme (IC50), was estimated from the graph plots of the dose–response curve for each sample concentration by GraphPad Prism software (San Diego, CA, USA).

#### 3.5.2. Anti-Elastase Assay

The anti-elastase assay was performed spectrophotometrically as previously reported by Kim et al. [74] with minor modifications. Elastase from pancreatic porcine was dissolved in sterile water to obtain a stock solution at a concentration of 3.33 mg/mL. N-succinyl-Ala–Ala–Ala–p-nitroanilide (AAAPVN) was used as a substrate, dissolved in Tris-HCL buffer (pH 8) at 1.6 mM. The samples in the concentration range of 1000–7.81 µg/mL were incubated at room temperature with the prepared elastase solution in the buffer for 15 min; then the synthetic substrate was added to the samples to start the reaction. The final mixture with a total volume 250 μL contained buffer, substrate (AAAPVN) 0.8 mM, 25 μg of test extract, and 1 μg/mL of PE. A positive control (EGCG) was used, while the negative control consisted of water. The absorbance was measured at 400 nm in a 96-well microtiter plate using a microplate reader (TECAN, Inc., Durham, NC, USA). The percentage of elastase inhibition was calculated according to (%) = [1 − (S/C) × 100], where “S” is the corrected absorbance of the tested samples, while “C” is the corrected absorbance of the controls (in the absence of a sample). The inhibitory concentration 50 (IC50) was estimated from the graph plots of the dose–response curves at each sample concentration by GraphPad Prism software (San Diego, CA, USA).

#### 3.5.3. Anti-Tyrosinase Assay

This assay was performed spectrophotometrically as previously described in the literature [75]. L-DOPA was used as a substrate. The reaction mixture in a total volume of 1000 μL contained 15 μL of mushroom tyrosinase (2500 U/mL), 685 μL of phosphate buffer (pH 6.5, 0.05 M), 100 μL of 5 mM L-DOPA, and 200 μL of the samples in the concentration range of 1000–7.81 µg/mL. The positive control kojic acid was used, whereas the negative control consisted of water. After the addition of the substrate (L-DOPA), the absorbance was measured at 475 nm using a microplate reader (TECAN, Inc., Durham, NC, USA); each measurement was carried out in triplicate. The percentage of tyrosinase inhibition was calculated according to (%) = [1 − (S/C) × 100], where “S” is the corrected absorbance of the tested sample, while “C” is the corrected absorbance of the controls (in the absence of a sample). The inhibitory concentration 50 (IC50) was estimated from the graph plots of the dose–response curves at each sample concentration by GraphPad Prism software (San Diego, CA, USA). IC50 is the concentration of the sample needed to inhibit 50% of tyrosinase activity under the used assay conditions.

#### 3.5.4. Anti-Hyaluronidase Assay

The hyaluronidase inhibitory assay was performed following the fluorimetric Morgan–Elson method reported by Reissig et al. [76] and modified by Takahashi et al. [59]. The reaction mixture in 2 mL test tubes contained 25 μL of calcium chloride (12.5 mM), 100 μL of hyaluronic acid substrate (1 mg/mL in 0.1 M acetate buffer; pH 3.5), 12.5 μL each of hyaluronidase (1.5 mg/mL), and the sample (2.8 mg/mL). The range of sample concentration for the hyaluronidase inhibition assay was 1000–7.81 µg/mL. Twenty-five microliters of KBO_2_ (0.8 M) was added to tubes, which were then placed in a water bath at 100 °C for 3 min, followed by cooling at room temperature. Then, 800 μL of DMAB (4 g DMAB in 5 mL 10 N HCl and 40 mL acetic acid) was added. Then, the tubes were incubated for 20 min at room temperature, and their contents were transferred to wells in a 96-well plate. Fluorescence was detected by a Tecan Infinite microplate reader (TECAN, Inc., Durham, NC, USA) at 545 nm excitation wavelength and 612 nm emission wavelength. The inhibitory concentration 50 (IC50), the concentration required to inhibit 50% of hyaluronidase activity under the used assay conditions, was estimated from the graph plot of the dose–response curves at each concentration using GraphPad Prism software (San Diego, CA, USA).

### 3.6. In Silico Studies

#### 3.6.1. In Silico Docking Studies on the Target Enzymes

The docking studies in the current work were performed using the platform of Vina autodock and M.G.L. tools 1.5.6 [77,78]. The crystal structures of the four potential targets, namely, collagenase, elastase, hyaluronidase, and tyrosinase were downloaded from the protein data bank using the following PDB IDs: 456c, 6qeo, 1fcv, and 5m8q, respectively. The four major compounds, vitamin E (*α*-tocopherol), *γ*-sitosterol, neophytadiene, and *β*-tocopherol, in addition to EGCG and kojic acid, were sketched using MOE builder and then energy-minimized under Amber12: EHT force field using a steepest descent algorithm [79,80]. The lowest energy conformations of each of the six compounds as well as of the four targets were saved in pdbqt format as a prerequisite for the Vina autodock. The applied docking approach was firstly validated by re-docking each co-crystalized ligand into the active site of its corresponding enzyme. This step was followed by RMSD calculation between the co-crystalized and the docked poses for each enzyme [81]. Finally, the four major compounds were docked in the binding site of each enzyme using the validated docking protocol. Besides, EGCG was docked in hyaluronidase, collagenase, and elastase, while kojic acid was docked in tyrosinase. Biovia Discovery Studio visualizer was implemented in docking analysis and used to generate the binding interaction images.

#### 3.6.2. In Silico Toxicity Study

In addition, an in silico toxicity study of the identified compounds was carried out. Any potential toxicities were predicted by Pro-toxII, while potential skin toxicity was predicted by Pred-Skin 3 using a Bayesian model [82,83].

### 3.7. Statistical Analysis

The performed assays were carried out in triplicates, and the values are expressed as mean ± SD. For the determination of the in vitro anti-collagenase, anti-tyrosinase, anti-elastase, anti-hyaluronidase activities, the (IC50) was estimated from the graph plots of the dose–response curves at each sample concentration by Graph Pad Prism software (San Diego, CA, USA). The IC50 is the concentration of the sample needed to inhibit 50% of the tested enzyme activity under the used assay conditions.

## 4. Conclusions

The present study investigated the volatile phytoconstituents and the biological activities of SSHF for the first time. The GC/MS analysis revealed that *α*-tocopherol, *γ*-sitosterol, neophytadiene, and *β*-tocopherol are the major compounds of the lipophilic fraction. In addition, SSHF showed significant anti-hyaluronidase, anti-collagenase, anti-tyrosinase activity, and low anti-elastase activity. The four major compounds achieved acceptable docking scores in the active sites of the target enzymes. The docking scores sometimes exceeded those of the reference compounds. In addition, the constituents of SSHF showed no skin toxicity in an in silico study. Based on these studies and previous reports, it can be concluded that the antiaging activities of SSHF may be attributed to its high content of vitamin E as well as to the synergistic action of all its constituents. Therefore, SSHF could be considered an excellent antiaging candidate that could be incorporated in pharmaceutical skin care products and cosmetics after further clinical trials.

## Figures and Tables

**Table 1:** Chemical profile of the prepared lipophilic extract from the leaves of *Stenocarpus sinuatus*.

Peak	Rt	Compound	Molecular Formula	RI_-exp_	RI_-lit_	Content (%)	Identification
1	31.54	3,7,11,15-Tetramethyl-2-hexadecene	C_20_H_40_	1811	1811	0.26%	MS, RI
2	31.67	Neophytadiene	C_20_H_38_	1817	1817	8.19%	MS, RI
3	32.58	7,11,15-Trimethyl-3-methylenehexadeca-1-ene	C_20_H_38_	1843	1844	3.07%	MS, RI
4	33.55	Hexadecanoic acid, methyl ester	C_17_H_34_O_2_	1907	1907	2.22%	MS, RI
5	36.89	9,12-Octadecadienoic acid, methyl ester	C_19_H_34_O_2_	2077	2076	1.79%	MS, RI
6	37.03	9,12,15-Octadecatrienoic acid, methyl ester, (*Z*, *Z*, *Z*)- (Linolenic acid, methyl ester)	C_19_H_32_O_2_	2084	2085	4.38%	MS, RI
7	37.23	Phytol	C_20_H_40_O	2095	2096	4.04%	MS, RI
8	48.92	Squalene	C_30_H_50_	2794	2790	1.03%	MS, RI
9	49.70	*α*-Tocospiro A	C_29_H_50_O_4_	2844	2855	0.64%	MS, RI
10	49.80	2-Methyloctacosane	C_29_H_60_	2851	2857	1.54%	MS, RI
11	51.97	*β*-Tocopherol	C_28_H_48_O_2_	3048	3043	6.07%	MS, RI
12	52.49	Hentriacontane	C_31_H_64_	3088	3100	1.64%	MS
13	53.27	Vitamin E (*α*-tocopherol)	C_29_H_50_O_2_	3146	3149	52.59%	MS, RI
14	54.84	Campesterol	C_28_H_48_O	3255	3193	0.66%	MS, RI
15	56.31	*γ*-Sitosterol	C_29_H_50_O	3346	3351	8.65%	MS, RI
Tocopherols						58.66%	
Acyclic diterpenes						11.52%	
Oxygenated diterpenes						4.04%	
Triterpenes						1.03%	
Sterols						9.31%	
Fatty acid methyl esters						8.39%	
Aliphatic alkanes						3.18%	
Others						0.64%	
Total identified						96.77%	

RI_exp_, Retention index, determined experimentally on an Rtx-5MS column; RI_lit_, published retention indices.

**Table 2:** Docking results regarding the main four isolated compounds and collagenase (456c), elastase (6qeo), hyaluronidase (1fcv), and tyrosinase (5M8Q).

Compound Name	Collagenase			Elastase			Hyaluronidase			Tyrosinase		
	Score	HB	Hydrophobic	Score	HB	Hydrophobic	Score	HB	Hydrophobic	Score	HB	Hydrophobic
*α*-Tocopherol	−9.02	-----	LEU185, LEU218, VAL219, LEU239, TYR244, PHE252, MET253	−6.00	HIS140, GLU141	HIS140, HIS223	−6.40	SER304	TYR55, TYR184, TYR227, TRP267, TRP301	−7.70	HIS377, HIS381	HIS215, PHE362, TYR369, HIS381, LEU382
*β*-Tocopherol	−8.68	PRO190	TYR176, LEU184, LEU185, HIS187, PRO193, HIS222, HIS226, HIS232	−6.20	-----	VAL137, HIS140, LEU197, ARG208, HIS223, HIS224	−6.40	SER304	TYR55, TYR184, TYR227, TRP301	−7.46	-----	HIS215, PHE362, TYR369, HIS377, HIS381, LEU382, VAL391
Neophytadiene	−7.83	-----	LEU184, LEU185, LEU218, VAL219, HIS222, LEU239, PHE252, MET252	−5.20	-----	LEU132, VAL137, HIS140, ILE190, LEU197, ARG208, HIS223, HIS224	−5.40	-----	TYR55, TRP184, TYR227, TRP301	−7.13	-----	HIS215, TYR348, LEU382, VAL391, PRO431, HIS434
*γ*-Sitosterol	−7.66	-----	TYR179, LEU184, TYR185, HIS187, PHE189, PRO193, HIS222, HIS232	−6.10	-----	PHE129, LEU132, LEU197, ARG208, HIS223	−6.90	-----	TYR55, TYR184, TYR227, TRP226, TRP301	−6.84	-----	HIS215, PHE362, TYR369, HIS377, HIS381, LEU382, VAL391
EGCG	−8.19	ALA186, VAL219, GLU223, HIS226, PHE241, ILE243	VLA219, HIS222	−8.80	GLU141, ARG198, ASP206, ASP221, HIS223	HIS140, LEU197, ARG198	−8.9	ASP56, ASP111, TRP301, SER304	ASP111, GLU113, TRP301			
Kojic acid										−4.62	HIS377, SER394	HIS377, SER394

**Table 3:** In silico toxicity study of compounds identified in SSHF.

Compound Name	lD_50_ as Predicted by Pro-toxII	Any Potential Toxicity as Predicted by Pro-toxII	Potential Skin Toxicity as Computed by Pred-Skin 3 Using Bayesian Model	% Probability
3,7,11,15-Tetramethyl-2-hexadecene	5000 mg/kg	none	None	95
Neophytadiene	5050 mg/kg	none	None	89
Hexadecanoic acid, methyl ester	5000 mg/kg	none	None	92
9,12-Octadecadienoic acid, methyl ester	20,000 mg/kg	none	None	95
9,12,15-Octadecatrienoic acid, methyl ester (Linolenic acid, methyl ester)	20,000 mg/kg	none	None	99
Phytol	5000 mg/kg	none	None	92
Squalene	5000 mg/kg	none	None	95
*α*-Tocospiro A	300 mg/kg	cytotoxic	None	88
2-Methyloctacosane	750 mg/kg	none	None	96
*β*-Tocopherol	500 mg/kg	none	None	90
Hentriacontane	750 mg/kg	none	None	98
Vitamin E (*α*-tocopherol)	5000 mg/kg	none	None	89
Campesterol	890 mg/kg	none	None	97
*γ*-Sitosterol	890 mg/Kg	none	None	98

## Data Availability

Data are available upon request from the first author.

## References

[1] Yagi M., Takabe W., Ishizaki K., Yonei Y. (2017). The glycative stress and skin aging, and its countermeasure. Fragr. J..

[2] Ganceviciene R., Liakou A.I., Theodoridis A., Makrantonaki E., Zouboulis C.C. (2012). Skin anti-aging strategies. Derm.-Endocrinol..

[3] Cavinato M., Waltenberger B., Baraldo G., Grade C.V.C., Stuppner H., Jansen-Duerr P. (2017). Plant extracts and natural compounds used against UVB-induced photoaging. Biogerontology.

[4] Banglao W., Thongmee A., Sukplang P., Wanakhachornkrai O. (2020). Determination of antioxidant, anti-aging and cytotoxicity activity of the essential oils from *Cinnamomum zeylanicum*. J. Microbiol. Biotechnol. Food Sci..

[5] Fayad S., Morin P., Nehmé R. (2017). Use of chromatographic and electrophoretic tools for assaying elastase, collagenase, hyaluronidase, and tyrosinase activity. J. Chromatogr. A.

[6] Abd Razak D.L., Jamaluddin A., Abd Rashid N.Y., Sani N.A., Abdul Manan M. (2020). Assessment of cosmeceutical potentials of selected mushroom fruitbody extracts through evaluation of antioxidant, anti-hyaluronidase and anti-tyrosinase activity. J.

[7] Mostafa N.M., Mostafa A.M., Ashour M.L., Elhady S.S. (2021). Neuroprotective effects of black pepper cold-pressed oil on scopolamine-induced oxidative stress and memory impairment in rats. Antioxidants.

[8] El-Nashar H.A.S., Mostafa N.M., Eldahshan O.A., Singab A.N.B. (2020). A new antidiabetic and anti-inflammatory biflavonoid from *Schinus polygama* (Cav.) Cabrera leaves. Nat. Prod. Res..

[9] Abdallah S.H., Mostafa N.M., Mohamed M.A., Nada A.S., Singab A.N.B. (2021). UPLC-ESI-MS/MS profiling and hepatoprotective activities of *Stevia* leaves extract, butanol fraction and stevioside against radiation-induced toxicity in rats. Nat. Prod. Res..

[10] Al-Madhagy S.A., Mostafa N.M., Youssef F.S., Awad G.E.A., Eldahshan O.A., Singab A.N.B. (2019). Metabolic profiling of a polyphenolic-rich fraction of *Coccinia grandis* leaves using LC-ESI-MS/MS and in vivo validation of its antimicrobial and wound healing activities. Food Funct..

[11] Mostafa N.M., Abd El-Ghffar E.A., Hegazy H.G., Eldahshan O.A. (2018). New methoxyflavone from *Casimiroa sapota* and the biological activities of its leaves extract against lead acetate induced hepatotoxicity in rats. Chem. Biodivers..

[12] Shahat E.A., Bakr R.O., Eldahshan O.A., Ayoub N.A. (2017). Chemical composition and biological activities of the essential oil from leaves and flowers of *Pulicaria incisa* sub. candolleana (Family Asteraceae). Chem. Biodivers..

[13] Singab A.N.B., Mostafa N.M., Eldahshan O.A., Ashour M.L., Wink M. (2014). Profile of volatile components of hydrodistilled and extracted leaves of *Jacaranda acutifolia* and their antimicrobial activity against foodborne pathogens. Nat. Prod. Commun..

[14] Todirascu-Ciornea E., El-Nashar H.A.S., Mostafa N.M., Eldahshan O.A., Boiangiu R.S., Dumitru G., Hritcu L., Singab A.N.B. (2019). *Schinus terebinthifolius* essential oil attenuates scopolamine-induced memory deficits via cholinergic modulation and antioxidant properties in a zebrafish model. Evid.-Based Complement. Altern. Med..

[15] Faheem S.A., Saeed N.M., El-Naga R.N., Ayoub I.M., Azab S.S. (2020). Hepatoprotective effect of cranberry nutraceutical extract in non-alcoholic fatty liver model in rats: Impact on insulin resistance and Nrf-2 expression. Front. Pharmacol..

[16] Divya G., Albert A., Singab A.N.B., Ayoub I.M., Al-Sayed E., Paul E., Manoharan K., Saso L., Selvam G.S. (2021). Renoprotective effect of tectorigenin glycosides isolated from *Iris spuria* L. (Zeal) against hyperoxaluria and hyperglycemia in NRK-49Fcells. Nat. Prod. Res..

[17] Ayoub I.M., George M.Y., Menze E.T., Mahmoud M., Botros M., Essam M., Ashmawy I., Shendi P., Hany A., Galal M. (2022). Insights into the neuroprotective effects of *Salvia officinalis* L. and *Salvia microphylla* Kunth in the memory impairment rat model. Food Funct..

[18] Elkady W.M., Ayoub I.M., Abdel-Mottaleb Y., ElShafie M.F., Wink M. (2020). *Euryops pectinatus* L. Flower extract inhibits P-glycoprotein and reverses multi-drug resistance in cancer cells: A mechanistic study. Molecules.

[19] Brinza I., Ayoub I.M., Eldahshan O.A., Hritcu L. (2021). Baicalein 5,6-dimethyl ether prevents memory deficits in the scopolamine zebrafish model by regulating cholinergic and antioxidant systems. Plants.

[20] Younis I.Y., Eldahshan O.A., Abdel-Aziz M.A., Ali Z.Y. (2021). Green synthesis of magnesium nanoparticles mediated from *Rosa floribunda charisma* extract and its antioxidant, antiaging and antibiofilm activities. Sci. Rep..

[21] Amuka O., Machocho A.K., Okemo P.O., Mbugua P.K. (2015). Antifungal and antibacterial activity of crude stem bark extracts’ of *Bersama abysinicca* Verdc. and *Faurea saligna* Harr. Res. J. Med. Plant.

[22] Deans B.J., Kilah N.L., Bissember A.C., Smith J.A., Jordan G.J. (2018). Arbutin derivatives isolated from ancient Proteaceae: Potential phytochemical markers present in Bellendena, Cenarrhenes, and Persoonia Genera. J. Nat. Prod..

[23] Fiorito S., Genovese S., Epifano F., Mathieu V., Kiss R., Taddeo V.A. (2016). Cytotoxic activity of lomatiol and 7-(3′-Hydroxymethyl-3′-methylallyloxy)coumarin. Nat. Prod. Commun..

[24] Giang P.M., Thao D.T., Nga N.T., Van Trung B., Anh D.H., Viet P.H. (2019). Evaluation of the antioxidant, hepatoprotective, and anti-inflammatory activities of bisresorcinol isolated from the trunk of *Heliciopsis Terminalis*. Pharm. Chem. J..

[25] Vinueza D., Yanza K., Tacchini M., Grandini A., Sacchetti G., Chiurato M.A., Guerrini A. (2018). Flavonoids in Ecuadorian *Oreocallis grandiflora* (Lam.) R. Br.: Perspectives of use of this species as a food supplement. Evid.-Based Complement. Altern. Med..

[26] Wang H., Leach D.N., Thomas M.C., Blanksby S.J., Forster P.I., Waterman P.G. (2011). Bisresorcinol derivatives from *Grevillea glauca*. Helv. Chim. Acta.

[27] Yang F., Zhao H., Carroll A.R. (2017). Tropane alkaloids from the Australian plant *Triunia montana* (Proteaceae). Tetrahedron Lett..

[28] Monzote L., Piñón A., Setzer W.N. (2014). Antileishmanial potential of tropical rainforest plant extracts. Medicines.

[29] Tong J., Zhou Z., Qi W., Jiang S., Yang B., Zhong Z., Jia Y., Li X., Xiong L., Nie L. (2019). Antidepressant effect of helicid in chronic unpredictable mild stress model in rats. Int. Immunopharmacol..

[30] El Hawary S.S., Abubaker M., Abd El-Kader E.M., Mahrous E.A. (2020). Phytochemical constituents and anti-tyrosinase activity of *Macadamia integrifolia* leaves extract. Nat. Prod. Res..

[31] Abubaker M., El Hawary S.S., Mahrous E.A., Abd El-Kader E.M. (2017). Study of Nutritional contents of *Macadamia integrifolia* Maiden & Betche leaves, kernel and pericarp cultivated in Egypt. Int. J. Pharmacogn. Phytochem. Res..

[32] Wall M.M. (2010). Functional lipid characteristics, oxidative stability, and antioxidant activity of macadamia nut (*Macadamia integrifolia*) cultivars. Food Chem..

[33] Kamagaju L., Morandini R., Bizuru E., Nyetera P., Nduwayezu J.B., Stevigny C., Ghanem G., Duez P. (2013). Tyrosinase modulation by five Rwandese herbal medicines traditionally used for skin treatment. J. Ethnopharmacol..

[34] Chuang T.-H., Chan H.-H., Wu T.-S., Li C.-F. (2011). Chemical constituents and biological studies of the leaves of *Grevillea robusta*. Molecules.

[35] Vinueza D., Cajamarca D., Acosta K., Pilco G. (2018). Oreocallis grandiflora photoprotective effect against ultraviolet B radiation-induced cell death. Asian J. Pharm. Clin. Res..

[36] Mock J., Murphy S.T., Ritchie E., Taylor W.C. (1973). Chemical studies of the Proteaceae. VI. Two naphthoquinones from *Stenocarpus salignus*. Aust. J. Chem..

[37] Dorey J.B. (2021). Missing for almost 100 years: The rare and potentially threatened bee, *Pharohylaeus lactiferus* (Hymenoptera, Colletidae). J. Hymenopt. Res..

[38] Kerdpol K., Nutho B., Krusong K., Poo-arporn R.P., Rungrotmongkol T., Hannongbua S. (2021). Encapsulation of α-tocopherol in large-ring cyclodextrin containing 26 α-D-glucopyranose units: A molecular dynamics study. J. Mol. Liq..

[39] Bruno R.S., Mah E., Vitamin E. (2014). Reference Module in Biomedical Sciences.

[40] Ching L.S., Mohamed S. (2001). Alpha-tocopherol content in 62 edible tropical plants. J. Agric. Food Chem..

[41] García M.F., Vergara C.E., Forero-Doria O., Guzman L., del Carmen Perez-Camino M. (2019). Chemical evaluation and thermal behavior of Chilean hazelnut oil (*Gevuina avellana* Mol) a comparative study with extra virgin olive oil. Eur. Food Res. Technol..

[42] Medel F., Nunez R., Medel G., Palma H., Manquian N., Fuentes R. (2009). Fractions of vitamin E (tocotrienols and tocopherols) in nut oil of *Gevuina avellana* Mol. Acta Hortic..

[43] Rattanawiwatpong P., Wanitphakdeedecha R., Bumrungpert A., Maiprasert M. (2020). Anti-aging and brightening effects of a topical treatment containing vitamin C, vitamin E, and raspberry leaf cell culture extract: A split-face, randomized controlled trial. J. Cosmet. Dermatol..

[44] Villareal M.O., Kume S., Bourhim T., Bakhtaoui F.Z., Kashiwagi K., Han J., Gadhi C., Isoda H. (2013). Activation of MITF by argan oil leads to the inhibition of the tyrosinase and dopachrome tautomerase expressions in B16 murine melanoma cells. Evid.-Based Complement. Altern. Med..

[45] Ricciarelli R., Maroni P., Özer N., Zingg J.-M., Azzi A. (1999). Age-dependent increase of collagenase expression can be reduced by α-tocopherol via protein kinase C inhibition. Free. Radic. Biol. Med..

[46] Heo S.-I., Jung M.-J., Kim M.-K., Wang M.-H. (2007). Antioxidative activities and tyrosinase inhibitory effects of Korean medicinal plants. J. Appl. Biol. Chem..

[47] Yenilmez E., Başaran E., Yazan Y. (2011). Release characteristics of vitamin E incorporated chitosan microspheres and in vitro–in vivo evaluation for topical application. Carbohydr. Polym..

[48] Poljšak N., Kočevar Glavač N. (2021). Tilia sp. Seed Oil—Composition, antioxidant activity and potential use. Appl. Sci..

[49] Doering T., Holtkötter O., Schlotmann K., Jassoy C., Petersohn D., Wadle A., Waldmann-Laue M. (2005). Cutaneous restructuration by apple seed phytosterols: From DNA chip analysis to morphological alterations. Int. J. Cosmet. Sci..

[50] Edmond M.P., Mostafa N.M., El-Shazly M., Singab A.N.B. (2021). Two clerodane diterpenes isolated from *Polyalthia longifolia* leaves: Comparative structural features, anti-histaminic and anti-*Helicobacter pylori* activities. Nat. Prod. Res..

[51] Mostafa N.M., Edmond M.P., El-Shazly M., Fahmy H.A., Sherif N.H., Singab A.N.B. (2021). Phytoconstituents and renoprotective effect of *Polyalthia longifolia* leaves extract on radiation-induced nephritis in rats via TGF-β/smad pathway. Nat. Prod. Res..

[52] Jeong S.H. (2018). Inhibitory effect of phytol on cellular senescence. Biomed. Dermatol..

[53] Islam M.T., Ali E.S., Uddin S.J., Shaw S., Islam M.A., Ahmed M.I., Shill M.C., Karmakar U.K., Yarla N.S., Khan I.N. (2018). Phytol: A review of biomedical activities. Food Chem. Toxicol..

[54] Yasmeen S., Gupta P. (2019). Interaction of selected terpenoids from *Dalbergia sissoo* with catalytic domain of matrix metalloproteinase-1: An in silico assessment of their anti-wrinkling potential. Bioinform. Biol. Insights.

[55] Temin P. (2016). GC- MS Analysis of *Mussaenda roxburghii* Hk.f.: A folk food plant used among tribes of Arunachal Pradesh, India. Pharmacogn. J..

[56] Ak G., Zengin G., Ceylan R., Fawzi Mahomoodally M., Jugreet S., Mollica A., Stefanucci A. (2021). Chemical composition and biological activities of essential oils from *Calendula officinalis* L. flowers and leaves. Flavour Fragr. J..

[57] Mahalakashmi R., Thangapandian V. (2019). Gas chromatography and mass spectrometry analysis of bioactive constituents of *Maytenus heyneana* (Roth) Roju & Babu (Celestraceae). J. Pharmacogn. Phytochem..

[58] Mostafa N.M., Ashour M.L., Eldahshan O.A., Singab A.N.B. (2016). Cytotoxic activity and molecular docking of a novel biflavonoid isolated from *Jacaranda acutifolia* (Bignoniaceae). Nat. Prod. Res..

[59] Moussa A.Y., Mostafa N.M., Singab A.N.B. (2020). Pulchranin A: First report of isolation from an endophytic fungus and its inhibitory activity on cyclin dependent kinases. Nat. Prod. Res..

[60] Ashmawy A., Mostafa N., Eldahshan O. (2019). GC/MS analysis and molecular profiling of lemon volatile oil against breast cancer. J. Essent. Oil Bear. Plants.

[61] Elhawary E.A., Mostafa N.M., Labib R.M., Singab A.N. (2021). Metabolomic profiles of essential oils from selected rosa varieties and their antimicrobial activities. Plants.

[62] El-Nashar H.A.S., Mostafa N.M., El-Badry M.A., Eldahshan O.A., Singab A.N.B. (2020). Chemical composition, antimicrobial and cytotoxic activities of essential oils from *Schinus polygamus* (Cav.) cabrera leaf and bark grown in Egypt. Nat. Prod. Res..

[63] Ashmawy A.M., Ayoub I.M., Eldahshan O.A. (2020). Chemical composition, cytotoxicity and molecular profiling of *Cordia africana* Lam. on human breast cancer cell line. Nat. Prod. Res..

[64] Labib R.M., Ayoub I.M., Michel H.E., Mehanny M., Kamil V., Hany M., Magdy M., Moataz A., Maged B., Mohamed A. (2019). Appraisal on the wound healing potential of *Melaleuca alternifolia* and *Rosmarinus officinalis* L. essential oil-loaded chitosan topical preparations. PLoS ONE.

[65] Gad H., Al-Sayed E., Ayoub I. (2021). Phytochemical discrimination of *Pinus* species based on GC–MS and ATR-IR analyses and their impact on *Helicobacter pylori*. Phytochem. Anal..

[66] Gad H.A., Ayoub I.M., Wink M. (2019). Phytochemical profiling and seasonal variation of essential oils of three *Callistemon* species cultivated in Egypt. PLoS ONE.

[67] Ayoub N., Singab A.N., Mostafa N., Schultze W. (2010). Volatile constituents of leaves of *Ficus carica* Linn. grown in Egypt. J. Essent. Oil Bear. Plants.

[68] Ayoub I.M., Korinek M., El-Shazly M., Wetterauer B., El-Beshbishy H.A., Hwang T.-L., Chen B.-H., Chang F.-R., Wink M., Singab A.N.B. (2021). Anti-allergic, anti-inflammatory, and anti-hyperglycemic activity of *Chasmanthe aethiopica* leaf extract and its profiling using LC/MS and GLC/MS. Plants.

[69] Korany D.A., Ayoub I.M., Labib R.M., El-Ahmady S.H., Singab A.N.B. (2021). The impact of seasonal variation on the volatile profile of leaves and stems of *Brownea grandiceps* (Jacq.) with evaluation of their anti-mycobacterial and anti-inflammatory activities. S. Afr. J. Bot..

[70] Thabet A.A., Ayoub I.M., Youssef F.S., Al Sayed E., Singab A.N.B. (2021). Essential oils from the leaves and flowers of *Leucophyllum frutescens* (Scrophulariaceae): Phytochemical analysis and inhibitory effects against elastase and collagenase in vitro. Nat. Prod. Res..

[71] Azab S.S., Abdel Jaleel G.A., Eldahshan O.A. (2017). Anti-inflammatory and gastroprotective potential of leaf essential oil of *Cinnamomum glanduliferum* in ethanol-induced rat experimental gastritis. Pharm. Biol..

[72] Brinza I., Abd-Alkhalek A.M., El-Raey M.A., Boiangiu R.S., Eldahshan O.A., Hritcu L. (2020). Ameliorative effects of rhoifolin in scopolamine-induced amnesic zebrafish (Danio rerio) model. Antioxidants.

[73] Thring T.S.A., Hili P., Naughton D.P. (2009). Anti-collagenase, anti-elastase and anti-oxidant activities of extracts from 21 plants. BMC Complementary Altern. Med..

[74] Kim Y.-J., Uyama H., Kobayashi S. (2004). Inhibition effects of (+)-catechin-aldehyde polycondensates on proteinases causing proteolytic degradation of extracellular matrix. Biochem. Biophys. Res. Commun..

[75] Batubara I., Darusman L.K., Mitsunaga T., Rahminiwati M., Djauhari E. (2010). Potency of Indonesian medicinal plants as tyrosinase inhibitor and antioxidant agent. J. Biol. Sci..

[76] Reissig J.L., Strominger J.L., Leloir L.F. (1955). A modified colorimetric method for the estimation of N-acetylamino sugars. J. Biol. Chem..

[77] Sanner M.F. (1999). Python: A programming language for software integration and development. J. Mol. Graph. Model..

[78] Vina A. (2010). Improving the speed and accuracy of docking with a new scoring function, efficient optimization, and multithreading Trott, Oleg; Olson, Arthur J. J. Comput. Chem..

[79] Case D.A., Cheatham Iii T.E., Darden T., Gohlke H., Luo R., Merz K.M., Onufriev A., Simmerling C., Wang B., Woods R.J. (2005). The Amber biomolecular simulation programs. J. Comput. Chem..

[80] Vilar S., Cozza G., Moro S. (2008). Medicinal chemistry and the molecular operating environment (MOE): Application of QSAR and molecular docking to drug discovery. Curr. Top. Med. Chem..

[81] Elsayed Z.M., Eldehna W.M., Abdel-Aziz M.M., El Hassab M.A., Elkaeed E.B., Al-Warhi T., Abdel-Aziz H.A., Abou-Seri S.M., Mohammed E.R. (2021). Development of novel isatin–nicotinohydrazide hybrids with potent activity against susceptible/resistant *Mycobacterium tuberculosis* and bronchitis causing–bacteria. J. Enzym. Inhib. Med. Chem..

[82] Banerjee P., Dehnbostel F.O., Preissner R. (2018). Prediction is a balancing act: Importance of sampling methods to balance sensitivity and specificity of predictive models based on imbalanced chemical data sets. Front. Chem..

[83] Braga R.C., Alves V.M., Muratov E.N., Strickland J., Kleinstreuer N., Trospsha A., Andrade C.H. (2017). Pred-skin: A fast and reliable web application to assess skin sensitization effect of chemicals. J. Chem. Inf. Model..

